# Honeybush Extracts (*Cyclopia* spp.) Rescue Mitochondrial Functions and Bioenergetics against Oxidative Injury

**DOI:** 10.1155/2020/1948602

**Published:** 2020-08-07

**Authors:** Anastasia Agapouda, Veronika Butterweck, Matthias Hamburger, Dalene de Beer, Elizabeth Joubert, Anne Eckert

**Affiliations:** ^1^University of Basel, Transfaculty Research Platform, Molecular and Cognitive Neuroscience, Neurobiology Lab for Brain Aging and Mental Health, Basel, Switzerland; ^2^Psychiatric University Clinics, Basel, Switzerland; ^3^University of Applied Sciences and Arts Northwestern Switzerland (FHNW), School of Life Sciences, Institute of Pharmaceutical Technology, Gründenstrasse 40, 4132 Muttenz, Switzerland; ^4^Division of Pharmaceutical Biology, Department of Pharmaceutical Sciences, University of Basel, Basel, Switzerland; ^5^Plant Bioactives Group, Post-Harvest and Agro-Processing Technologies, Agricultural Research Council (ARC) Infruitec-Nietvoorbij, Private Bag X5026, Stellenbosch 7599, South Africa; ^6^Department of Food Science, Stellenbosch University, Private Bag X1, Matieland, Stellenbosch, South Africa

## Abstract

Mitochondrial dysfunction plays a major role not only in the pathogenesis of many oxidative stress or age-related diseases such as neurodegenerative as well as mental disorders but also in normal aging. There is evidence that oxidative stress and mitochondrial dysfunction are the most upstream and common events in the pathomechanisms of neurodegeneration. *Cyclopia* species are endemic South African plants and some have a long tradition of use as herbal tea, known as honeybush tea. Extracts of the tea are gaining more scientific attention due to their phenolic composition. In the present study, we tested not only the *in vitro* mitochondria-enhancing properties of honeybush extracts under physiological conditions but also their ameliorative properties under oxidative stress situations. Hot water and ethanolic extracts of *C. subternata*, *C. genistoides*, and *C. longifolia* were investigated. Pretreatment of human neuroblastoma SH-SY5Y cells with honeybush extracts, at a concentration range of 0.1-1 ng/ml, had a beneficial effect on bioenergetics as it increased ATP production, respiration, and mitochondrial membrane potential (MMP) after 24 hours under physiological conditions. The aqueous extracts of *C. subternata* and *C. genistoides*, in particular, showed a protective effect by rescuing the bioenergetic and mitochondrial deficits under oxidative stress conditions (400 *μ*M H_2_O_2_ for 3 hours). These findings indicate that honeybush extracts could constitute candidates for the prevention of oxidative stress with an impact on aging processes and age-related neurodegenerative disorders potentially leading to the development of a condition-specific nutraceutical.

## 1. Introduction

Reactive oxygen species (ROS) are oxygen-containing chemical entities of great reactivity that have been in the spotlight as a common feature in many diseases. They are involved in neurodegenerative and cardiovascular diseases, cancer, atherosclerosis, diabetes, and also in normal aging [1–4]. ROS include mainly superoxide anion radical (O_2_^·–^), hydrogen peroxide (H_2_O_2_), and the hydroxyl radical (OH^−^) of which superoxide anion and hydrogen peroxide are found in the most abundance in cells [5]. Mitochondria are organelles which are responsible for the majority of adenosine triphosphate (ATP) production through oxidative phosphorylation (OXPHOS) taking place at their electron transport chain (ETC). Neurons are high-energy demanding cells and thus are highly dependent on mitochondria in order to survive and function. However, mitochondria are also the epicenter of ROS production and metabolism [2, 6]. Despite an estimation of 31 existing ROS (mostly superoxide anion and H_2_O_2_) production sites in the entire cell versus 12 ROS emission sites in the mitochondria, the majority of cellular endogenous ROS are produced by mitochondria as by-products of OXPHOS [5, 7].

Exposure to oxygen is not only unavoidable but also vital and necessary for organism and cell survival and for energy production [5]. Mitochondrial ROS are mostly generated by complexes I and III of the ETC when leaking electrons that are provided by NADH or FADH_2_ react with oxygen. Interestingly, the two high-production sites releasing O_2_^·–^ and H_2_O_2_ directly into the intermembrane space are the enzyme sn-glycerol-3-phosphate dehydrogenase and complex III of the ETC [5]. As a result, the presence of ROS in the intermembrane space may cause depolarization of the membranes and hinder the free motion of electrons through complexes I-IV, thereby directly affecting the proton gradient and the mitochondrial membrane potential (MMP) and ultimately preventing the production of ATP [8].

As mitochondria are the main superoxide anion and hydrogen peroxide producers, they largely affect redox homeostasis [9]. For their protection, cells are equipped with antioxidant defense systems (superoxide dismutases, glutathione peroxidases, thioredoxin, catalase, and glutathione GSH) in order to fend off ROS [10–12]. The redox state of the cells is dynamic and depends on the production of ROS and the functionality of the antioxidant defense systems. At normal nonelevated concentrations, ROS act as signaling molecules and they participate in the regulation of senescence, cell death, and proliferation. When there is an overproduction of ROS, the antioxidant defense systems are overwhelmed and they are not able to diffuse them. Therefore, oxidative stress is the overaccumulation of ROS (mainly superoxide anion and H_2_O_2_) due to their overproduction or overburdened antioxidant defense systems [1, 5]. ROS react with and damage many cellular and mitochondrial biomolecules. Of note, they cause lipid peroxidation and membrane damage, protein misfolding, as well as DNA damage [3]. Mitochondrial DNA (mtDNA) is located in the matrix of mitochondria and encodes for 13 proteins which are structural components of the ETC. MtDNA is in very close proximity to the ROS production sites and is therefore directly affected and mutated, leading to faulty ETC components which leads back to impaired OXPHOS and more production of ROS [10, 13]. When the ROS levels surpass a certain threshold, then they become mitochondria-damaging and disease-causing agents [14]. Aging is characterized by an increase in ROS and a decrease in antioxidant defenses leading to mitochondrial damage and ultimately to cellular dysfunction, senescence, and apoptosis. Normal aging and neurodegenerative disorders have these characteristics in common although to a different extent. In neurodegeneration, the damaging effects are even more profound [3, 5, 9, 15].

Hydrogen peroxide, which is endogenously produced in mitochondria, is considered the ROS with the most impact on the fate of the cell. It can easily diffuse through membranes and has the greatest life span [9]. Therefore, hydrogen peroxide was used as an oxidative stressor in this study.

*Cyclopia* species, belonging to the Fabaceae family, are endemic to South Africa. Old records describe the traditional use of several species including *C. subternata*, *C. genistoides*, and *C. longifolia* as herbal teas [16]. At present, these *Cyclopia* species form the bulk of cultivated plant material supplementing plant material harvested in the wild and crucial to meet the growing demand of international markets. The main product is “fermented” (oxidised) honeybush tea, while the green (unoxidised) herbal tea is preferred for nutraceutical extract production due to a higher phenolic content and antioxidant capacity. The phenolic profile of honeybush varies qualitatively and quantitatively depending on the *Cyclopia* species. Major phenolic constituents belong to xanthone, benzophenone, flavanone, flavone, and dihydrochalcone subclasses [17]. Increased consumption and popularity of honeybush came along with increasing research interest in order to reveal new bioactivities and to examine its potential use as a nutraceutical and functional food [16, 18]. Quite predictably due to their phenolic composition, honeybush extracts have been shown to possess antioxidant activities which are of great importance and interest in the research of oxidative stress-related diseases [19–22]. Considering on the one hand the evidence of its antioxidant capacity and on the other hand the need for mitochondria-targeting antioxidant substances for use in the prevention of oxidative damage or the amelioration of increased oxidative stress levels, we hypothesized that honeybush could possess some beneficial mitochondria-enhancing properties. For this reason, this study is aimed at examining the protective effects of honeybush extracts against H_2_O_2_-induced oxidative stress in SH-SY5Y neuronal cells with a focus on mitochondria. To our knowledge, this is the first study that evaluates the effects of honeybush extracts on mitochondrial function in a neuronal cell model.

## 2. Materials and Methods

### 2.1. Chemicals and Reagents

Dulbecco's modified Eagle medium (DMEM), phosphate-buffered saline (PBS), fetal calf serum (FCS), Hanks' balanced salt solution (HBSS), penicillin/streptomycin, pyruvate, dihydrorhodamine 123 (DHR), 2′,7′-dichlorodihydrofluorescein diacetate (DCF), dihydroethidium (DHE), tetramethylrhodamine methyl ester (TMRM), gelatin, and H_2_O_2_ were from Sigma-Aldrich (St. Louis, MO, USA). MitoSOX and GlutaMAX were from Gibco Invitrogen (Waltham, MA, USA), ATPlite1step kit from PerkinElmer (Waltham, Massachusetts, USA), and XF Cell Mitostress kit from Seahorse Bioscience (North Billerica, MA, USA). Folin-Ciocalteau reagent was purchased from Merck (Darmstadt, Germany). Authentic reference standards (purity > 95%) for identification and quantification of phenolic compounds were obtained from Sigma-Aldrich (hesperidin), Extrasynthese (Genay, France; mangiferin, eriocitrin), Chemos (Regenstauf, Germany; isomangiferin), and Phytolab (Vestenbergsreuth, Germany; vicenin-2, 3-*β*-D-glucopyranosyliriflophenone). Compounds from the Plant Bioactives Group library included 3-*β*-D-glucopyranosyl-4-*O*-*β*-D-glucopyranosyliriflophenone, 3-*β*-D-glucopyranosylmaclurin and (2S)-5-*O*-[*α*-L-rhamnopyranosyl-(1 → 2)-*β*-D-glucopyranosyl] naringenin isolated from *C. genistoides*, and scolymoside and 3′,5′-di-*β*-D-glucopyranosylphloretin isolated from *C. subternata*. HPLC gradient grade “far UV” acetonitrile was supplied by Merck.

### 2.2. Plant Material and Extract Preparation

Harvesting of aerial parts (shoots and leaves) occurred in March 2017. *Cyclopia subternata* was harvested on Elsenburg research farm (-34.30267, 19.13809), while *C. longifolia* and *C. genistoides* were harvested on Nietvoorbij research farm (-33.90619, 18.87031), both located in the Western Cape Province of South Africa. The fresh plant material was mechanically cut into small pieces (<3 mm) and dried at 40°C in a cross-flow, temperature-controlled drying tunnel to a moisture content < 7% as for green honeybush tea production. The dried plant material was coarsely milled using a rotary mill equipped with a 1 mm sieve (Retsch, GmbH, Haan, Germany).

Hot water extracts were prepared from each batch of milled plant material by extracting 70 g plant material with 700 ml deionised water at 93°C for 30 min followed by filtration and freeze drying of the filtrate as previously described [23]. Similarly, 40% EtOH-water (v/v) extracts were prepared by extracting the milled plant material at 70°C for 30 min. Ethanol was removed under vacuum using rotary evaporation, and the remaining aqueous layer was freeze-dried. Prior to extraction using 70% EtOH-water (v/v), the plant material was subjected to exhaustive Soxhlet extraction with dichloromethane to remove chlorophyll. The defatted plant material was air-dried and further treated as for the 40% EtOH-water (v/v) extracts. The freeze-dried extracts (>15 g/extract) were coded, aliquoted into glass vials (for testing and retention samples), sealed, and stored under desiccation in the dark.

### 2.3. Quantification and Identification of Phenolic Compounds

The major phenolic compounds in the extracts were quantified using the respective species-specific validated HPLC-DAD method for *C. subternata* [23], *C. longifolia* [24], and *C. genistoides* [25]. Samples were dissolved in water or 10% DMSO and filtered using 0.45 *μ*m pore size PVDF syringe filters (Merck) for *C. subternata*, while 0.22 *μ*m pore size filters were used for *C. genistoides* and *C. longifolia*. Ascorbic acid was added to prevent compound degradation during analysis (final concentration ca 9 mg/ml). Peak areas at the appropriate wavelength together with external calibration curves were used for quantification (benzophenones, flavanones, and dihydrochalcones at 288 nm; xanthones and flavones at 320 nm). In cases where authentic reference standards were not available, quantification was in equivalents of a similar compound.

Total polyphenol content of extracts was determined using the Folin-Ciocalteau assay as adapted for microplate by Arthur et al. [26]. Values were expressed as g gallic acid equivalents per 100 g extract.

Extracts selected for further study after initial testing were also analyzed by LC-MS using a Waters Acquity ultra-performance liquid chromatography (UPLC) instrument coupled to a Synapt G2 quadrupole time-of-flight (Q-TOF) MS detector equipped with an electrospray ionization (ESI) source (Waters, Milford, USA). Mass calibration was performed using a sodium formate solution, and leucine enkephalin was used as the lockspray solution. Analysis was first performed in the MS^E^ mode with negative ionization: scanning range, 150–1500 am; capillary voltage, -2.5 kV; sampling cone voltage, 15.0 V; source temperature, 120°C; desolvation temperature, 275°C; cone gas flow (N_2_), 650 l/h; desolvation gas flow (N_2_), 50 l/h. For the MS/MS experiments, a collision energy of 30.0 V was used. Peaks were identified by comparing UV-Vis spectra, relative retention time, MS characteristics (molecular formula predicted by accurate mass), and MS/MS fragmentation spectra with those of authentic standards or literature data.

### 2.4. Cell Culture

The human neuroblastoma SH-SY5Y cell line was selected as our cellular model in this study as it is a well-established and widely used neuronal model in biochemical studies in general. The cell line behaves as human neuronal network in a dish and has been largely used in research as it expresses neuronal receptors. The SH-SY5Y cells were kept and grown at 37°C in a humidified incubator chamber under an atmosphere of 7.5% CO_2_ in DMEM supplemented with 10% (v/v) heat-inactivated FCS, 2 mM GlutaMAX, and 1% (v/v) penicillin/streptomycin. Cells were passaged 1-2 times per week, and the cells used for the experiments did not exceed passage 20. The cells were plated when they reached 80–90% confluence.

### 2.5. Treatment of Cells

Evaluation of ATP production was conducted on SH-SY5Y neuroblastoma cells to determine the potential toxic concentration range of the nine honeybush extracts. Two screenings were performed. Initially, aqueous, 70% ethanolic and 40% ethanolic extracts of the species *C. subternata*, *C. genistoides*, and *C. longifolia* were screened at a very broad concentration range of 0.1 ng/ml to 1 mg/ml (data not shown). Of note, all dry extracts were dissolved in DMSO for our experiments (final concentration of DMSO < 0.005%, no effect of the vehicle solution alone compared to the untreated condition). The first screening revealed that the extracts were not toxic for the neuroblastoma cells up to a concentration of 10 *μ*g/ml. According to the results of the first screening, the concentration range was reduced down to that of 0.1 ng/ml to 1 *μ*g/ml and the number of extracts was reduced from nine down to four (according to the capacity of the extracts in increasing the ATP levels of the cells) and a second screening cycle was performed. The screening was conducted by using an ATP detection assay (ATPlite 1step kit was from PerkinElmer). For the experiments, cells were plated and treated 1 day after plating for 24 h either with DMEM (untreated cells—control condition) or with a final concentration of 0.1 ng/ml to 1 *μ*g/ml of the extracts.

Because vehicle treatment was without any effect in our assays, we evaluated the effects of the honeybush extract concentrations in comparison to the untreated control condition in the following experiments. Cellular sensitivity of SH-SY5Y cells was confirmed by using the positive control estradiol as previously described in Grimm et al. 2014 [27].

Hydrogen peroxide (H_2_O_2_) which belongs to the reactive oxygen species produced by mitochondria was used as a stressor at the concentration 400 *μ*M which was able to decrease mitochondrial and cellular functions. The H_2_O_2_ concentration was selected based on screening experiments conducted on SH-SY5Y cells. For the stress experiments, cells were firstly pretreated for 24 h with the honeybush extracts and then treated for 3 h with 400 *μ*M H_2_O_2_. Each assay was conducted and repeated at least in triplicate.

### 2.6. ATP Levels

Total ATP content was determined using a bioluminescence assay (ATPlite 1step) according to the instructions of the manufacturer and as previously described [28–30]. Cells were plated in 6 replicates into white 96-well cell culture plates at a density of 1 × 10^4^ cells/well. The ATP was extracted from the cells upon lysis and it was transformed into light. The method measures the formation of light from ATP and luciferin catalyzed by the enzyme luciferase. The emitted light was linearly correlated to the ATP concentration and was measured using the multimode plate reader Cytation 3 (BioTek instruments, Winooski, Vermont, United States).

### 2.7. Determination of Mitochondrial Membrane Potential (MMP)

The MMP was measured using the fluorescent dye TMRM, since its transmembrane distribution depends on the MMP. As previously described [31, 32], the cells were plated in 6 replicates into black 96-well cell culture plates at a density of 1 × 10^4^ cells/well and were incubated with the dye at a concentration of 0.4 *μ*M for 20 min. After washing three times with HBSS, fluorescence was measured at 548 nm (excitation)/574 nm (emission), using a Cytation 3 multimode plate reader (BioTek instruments).

### 2.8. Mitochondrial Respiration

Mitochondrial respiration and cellular glycolysis were measured using the Seahorse Bioscience XF24 analyser as described before [28, 29, 33]. Briefly, XF24 cell culture microplates were coated with 0.1% gelatin and cells were plated at a density of 2.5 × 10^4^ cells/well in treatment medium (100 *μ*l) containing 1 g/l glucose, 4 mM pyruvate, and 10% FCS. After treatment with honeybush extracts for 24 h, the cells were washed once with PBS and then 500 *μ*l of assay medium (DMEM containing 1 g/l of glucose and 4 mM of pyruvate) was added to each well. The oxygen consumption rate (OCR) and extracellular acidification rate (ECAR) were measured concurrently under basal respiration. The data were extracted from the Seahorse XF24 software, and bioenergetic parameters (basal respiration, ATP production, maximal respiration, spare respiratory capacity, and glycolytic reserve) were calculated according to the guidelines of the manufacturer.

### 2.9. Determination of ROS Levels

Mitochondrial and cytosolic ROS levels and the specific levels of mitochondrial O_2_^·–^superoxide anion radicals and the total levels of O_2_^·–^ superoxide anion radicals levels were assessed using the fluorescent dyes dihydrorhodamine 123 (DHR), 2′,7′-dichlorodihydrofluorescein diacetate (DCF), the Red Mitochondrial Superoxide Indicator (MitoSOX), and dihydroethidium (DHE), respectively, as described before [30, 34]. SH-SY5Y cells were plated in 6 replicates into black 96-well cell culture plates at a density of 1 × 10^4^ cells/well. After treatment with honeybush extracts alone or after pretreatment with honeybush extracts, followed by treatment with H_2_O_2_, cells were treated with 10 *μ*M of one of the dyes: DCF, DHR, or DHE for 20 min or 5 *μ*M of MitoSOX for 90 min at room temperature in the dark on an orbital shaker. After washing the cells three times with HBSS, the formation of green fluorescent products triggered by DCF and DHR, respectively, was detected at 485 nm (excitation)/535 nm (emission). MitoSOX triggers the formation of red fluorescent products which were detected at 531 nm (excitation)/595 nm (emission). DHE, which is permeable to cells, is used as a total O_2_^·–^ superoxide anion detector as it is oxidised to the impermeable red fluorescent product ethidium, detected at 531 nm (excitation)/595 nm (emission). The intensity of fluorescence was proportional to mitochondrial ROS, cytosolic ROS, and O_2_^·–^ levels (total and mitochondrial). The fluorescence was measured using the Cytation 3 multimode plate reader.

### 2.10. Statistical Analysis

Data are given as the mean ± SEM. Statistical analyses were performed using GraphPad Prism software (version 5.02 for Windows, San Diego, California, USA). For statistical comparisons of more than two groups, one-way ANOVA was used, followed by a Dunnett's multiple comparison tests versus the control for physiological conditions and versus H_2_O_2_ for stress conditions. *P* < 0.05 was considered statistically significant.

## 3. Results

Two cycles of screenings were conducted with regard to the ability of each extract in increasing the ATP production of SH-SY5Y cells. The nine *Cyclopia* extracts produced by extraction of *C. subternata*, *C. genistoides*, and *C. longifolia* with hot water and two ethanol-water mixtures were screened (data not shown), and the four most promising extracts in terms of increased ATP production were selected for all subsequent experiments: the water extracts of all three *Cyclopia* species and the 70% ethanolic extract of *C. genistoides*.  Table 1 gives the content of the major phenolic compounds present in the selected extracts. Qualitative and quantitative differences in the phenolic profile are evident, notably the absence or presence of only trace levels of dihydrochalcones in *C. longifolia* and *C. genistoides* but substantial xanthone levels compared to *C. subternata*. Mangiferin followed by isomangiferin was the predominant compound in the *C. longifolia* and *C. genistoides* extracts. Scolymoside, a flavone rutinoside, followed by 3-*β*-D-glucopyranosyl-4-*O*-*β*-D-glucopyranosyliriflophenone, a benzophenone, was the main phenolic compound in *C. subternata* water extract. Scolymoside was not detected in the two *C. genistoides* extracts, but these extracts had substantially higher levels of the flavone di-glucoside, vicenin-2, compared to the *C. subternata* and *C. longifolia* extracts. Overall, the total phenolic content, based on the sum of individual phenolic compound content, was highest in the 70% EtOH-water extract of *C. genistoides* and lowest in the water extract of *C. subternata*. The total polyphenol content determined using the Folin-Ciocalteau assay was highest in the 70% EtOH-water extract of *C. genistoides* and lowest in the water extract of *C. longifolia* with similar values for the water extracts of *C. subternata* and *C. genistoides* (Table 1).

### 3.1. Honeybush Extracts Increase ATP Production under Physiological Conditions and under H_2_O_2_-Induced Stress

**Figure 1:**
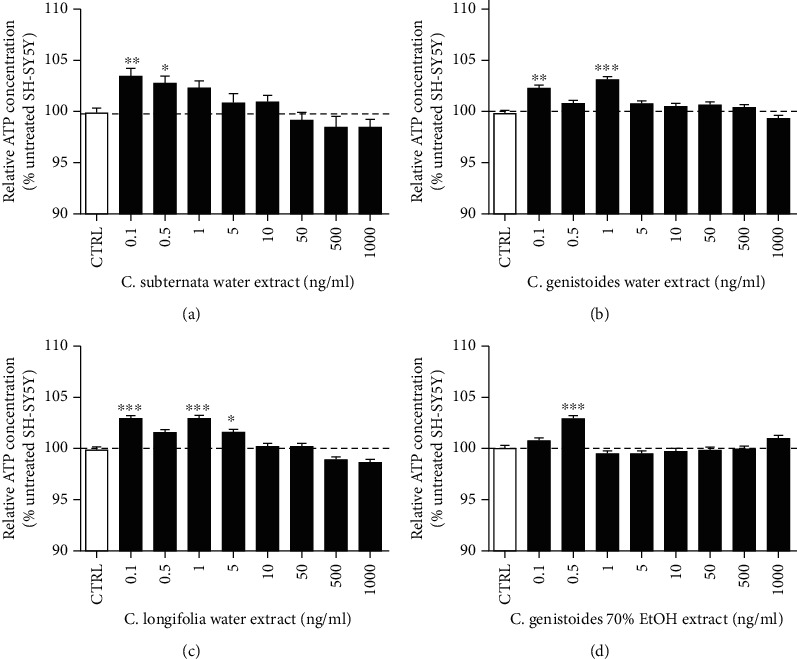
(a–d) The water extracts of *Cyclopia subternata*, *C. genistoides*, and *C. longifolia*, and the 70% ethanolic extract of *C. genistoides* significantly increased the ATP levels up to 4%. The cells were treated for 24 h with the extracts. Values represent as the mean ± SEM of three independent experiments and were normalized on the untreated (CTRL) group (=100%). One-way ANOVA and post hoc Dunnett's multiple comparison test versus CTRL cells. ^∗^*P* < 0.05; ^∗∗^*P* < 0.01; ^∗∗∗^*P* < 0.001.

ATP is the end product not only of mainly oxidative phosphorylation but also of glycolysis and is thus an indicator of mitochondrial and cellular viability and proper functioning. Therefore, we assessed the effect of the honeybush extracts on the ATP production of neuroblastoma cells. The concentration range of 0.1-1000 ng/ml for each extract was first tested under physiological conditions. The results indicated that the lower concentrations (0.1-1 ng/ml), but not the higher ones (50 ng/ml-100 mg/ml, data not shown), of the water extracts of the three *Cyclopia species* and of the 70% ethanolic extract of *C. genistoides* significantly increased ATP production up to 4% after treatment for 24 h under physiological conditions (Figures 1(a)–1(d)).

**Figure 2:**
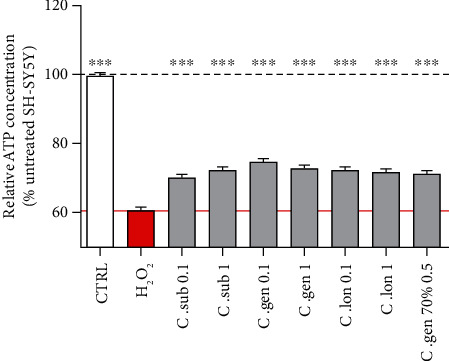
H_2_O_2_ treatment at 400 *μ*M for 3 h caused a 39.1% decrease in ATP production which was significantly increased up to 13.5% by a 24 h pretreatment with each of the extracts. The red bar represents the H_2_O_2_-treated cells, and the grey bars represent cells that were pretreated for 24 h with the indicated honeybush extract and then treated for 3 h with H_2_O_2_. Values represent as the mean ± SEM of three independent experiments and were normalized on the untreated group (=100%). One-way ANOVA and post hoc Dunnett's multiple comparison test versus H_2_O_2_-treated cells. ^∗^*P* < 0.05; ^∗∗^*P* < 0.01; ^∗∗∗^*P* < 0.001.

Regarding ATP levels under oxidative stress, H_2_O_2_ at 400 *μ*M caused a 39.1% decrease in ATP production. According to the experimental design under physiological conditions, we tested the same broad concentration range for each extract under oxidative stress (data not shown). Again, the concentrations 0.1 and 1 ng/ml significantly protected against oxidative stress. Therefore, these concentrations were used in the following oxidative stress experiments. The harmful effect of H_2_O_2_ was partially but significantly ameliorated by all the extracts up to 13.5% (Figure 2).

### 3.2. Honeybush Extracts Increase Mitochondrial Respiration under Physiological Conditions and under H_2_O_2_-Induced Stress

**Figure 3:**
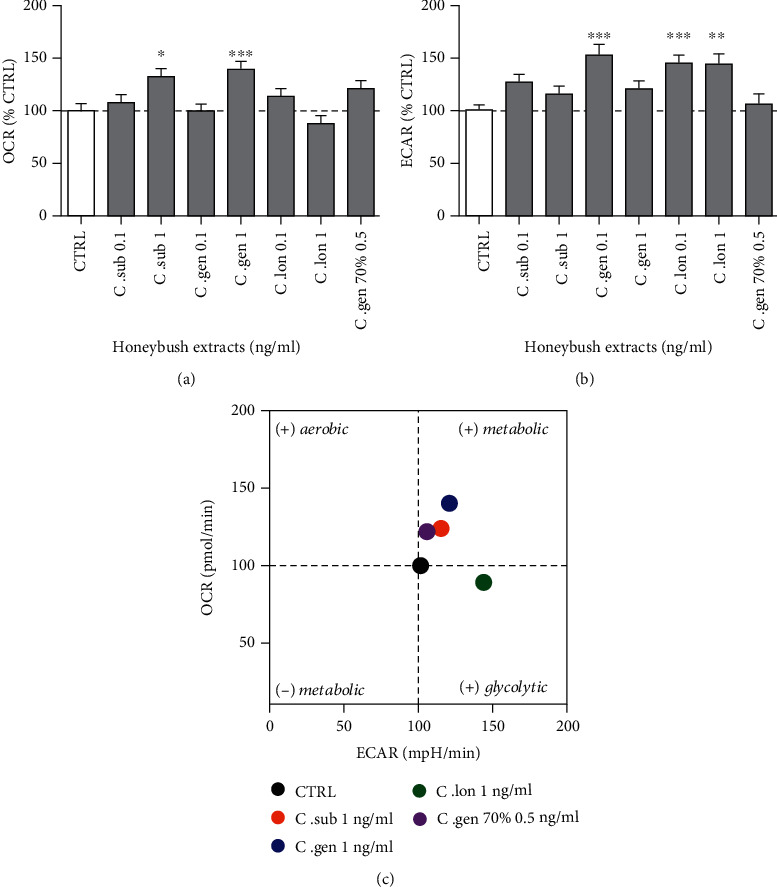
Respiration under physiological condition. (a) 24 h pretreatment with the water extracts of *C. subternata* and *C. genistoides* (both at 1 ng/ml) significantly increased the oxygen consumption rate of the cells and therefore the respiration. (b) The water extracts of *C. genistoides* (0.1 ng/ml) and *C. longifolia* (0.1 and 1 ng/ml) significantly increased the glycolysis in the SH-SY5Y cells. (c) Energy map created after correlation of the OCR (respiration—*y*-axis) with the ECAR (glycolysis—*x*-axis). The aqueous extracts of *C. subternata* and *C. genistoides* acted on respiration (displayed as + metabolic in the figure), while the water extract of *C. longifolia* increased the glycolytic activity. Values represent as the mean ± SEM of three independent experiments and were normalized on the comparison test versus H_2_O_2_-treated cells. ^∗^*P* < 0.05; ^∗∗^*P* < 0.01; ^∗∗∗^*P* < 0.001.

Mitochondria consume oxygen to perform respiration and oxidative phosphorylation. Thus, for an assessment of mitochondrial respiration, the oxygen consumption rate of the cells was measured live under basal conditions. The results indicated that the water extracts of *C. subternata* and *C. genistoides* and the 70% ethanolic extract of *C. genistoides* increased the respiration under physiological conditions at baseline. However, upon closer analysis of data, it was found that only the water extracts of *C. subternata* and *C. genistoides* at 1 ng/ml significantly increased the respiration by 33.2% and 40.7%, respectively (Figure 3(a)). The extracts that significantly increased the other pathway leading to the production of ATP, glycolysis, were *C. genistoides* (1 ng/ml) and *C. longifolia* (at 0.1 and 1 ng/ml). This increase was up to 51.7% (Figure 3(b)). Upon correlation of the respiration with the glycolysis, an “energy map” was obtained (Figure 3(c)) which allows a visual representation of where each individual extract acted. Thus, *C. subternata* and *C. genistoides* increased the oxygen consumption rate of the cells (respiration), while *C. longifolia* increased the glycolysis.

**Figure 4:**
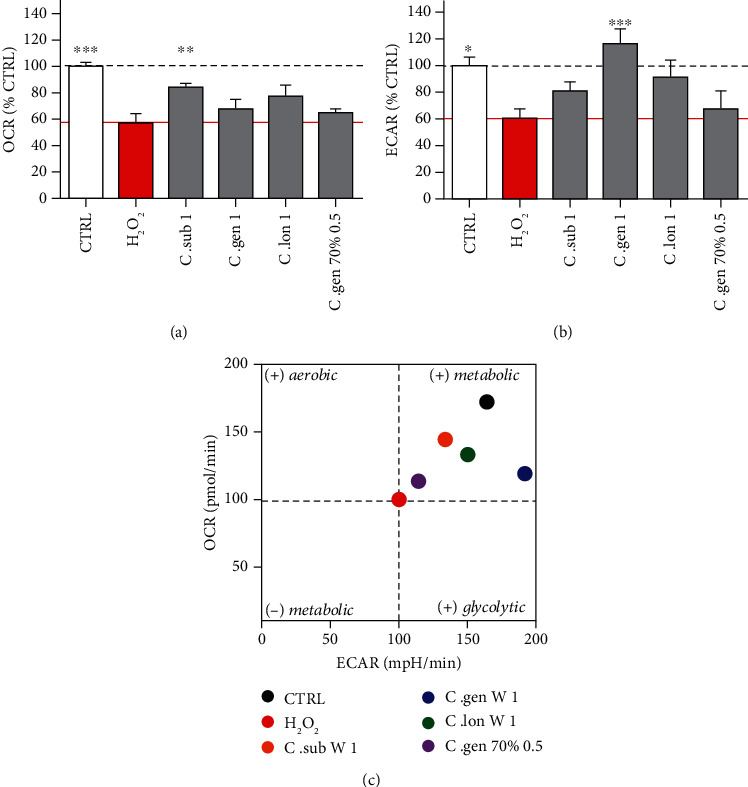
Respiration under oxidative stress condition. (a) 3 h treatment with H_2_O_2_ reduced the oxygen consumption rate by 41.7% (red bars). 24 h pretreatment with the water extract of *C. subternata* (at 1 ng/ml) significantly ameliorated the oxygen consumption rate of the cells and therefore the respiration (grey bars). (b) 3 h treatment with H_2_O_2_ reduced the glycolysis by 38.9% (red bars). The aqueous extract of *C. genistoides* (1 ng/ml) significantly increased the glycolysis in SH-SY5Y cells. The red bar represents the H_2_O_2_-treated cells, and the grey bars represent cells that were pretreated for 24 h with the indicated honeybush extract and then treated for 3 h with H_2_O_2_. (c) Energy map created after correlation of the OCR (respiration—*y*-axis) with the ECAR (glycolysis—*x*-axis). This map helps in visually recognizing whether an extract predominantly increased the respiration (displayed as + metabolic in the figure) or the glycolytic activity compared to the H_2_O_2_-treated cells. Values represent as the mean ± SEM of three independent experiments and were normalized on the untreated group (=100%). One-way ANOVA and post hoc Dunnett's multiple comparison test versus H_2_O_2_-treated cells. ^∗^*P* < 0.05; ^∗∗^*P* < 0.01; ^∗∗∗^*P* < 0.001.

H_2_O_2_ caused a significant decrease of 41.7% in respiration (Figure 4(a), red bar). All extracts increased the oxygen consumption rate, bringing it closer to the levels of the untreated cells. However, only the water extract of *C. subternata* was able to significantly enhance respiration at baseline (increase of 25.9%) (Figure 4(a)). Regarding glycolysis, H_2_O_2_ caused a significant decrease of 38.9% which was completely rescued by the water extract of *C. genistoides* (1 ng/ml) (Figure 4(b)). The “energy map” confirmed that the most effective extract in rescuing the respiration under H_2_O_2_ stress was the aqueous extract of *C. subternata* (Figure 4(c)).

### 3.3. Honeybush Extracts Increase Mitochondrial Membrane Potential (MMP) under Physiological Conditions and under H_2_O_2_-Induced Stress

**Figure 5:**
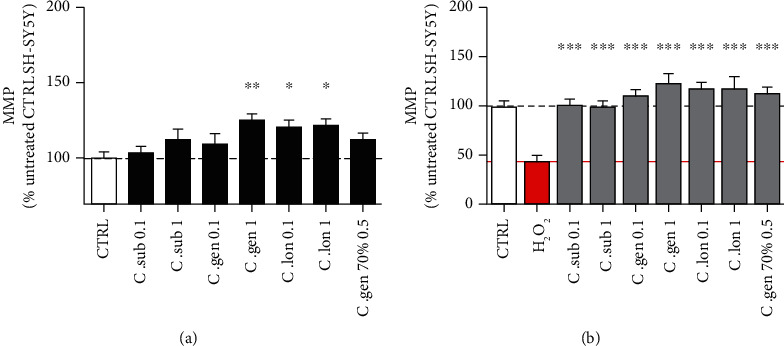
(a) Honeybush extracts significantly increased the mitochondrial membrane potential (MMP) up to 24% under physiological conditions (black bars). (b) 3 h treatments with H_2_O_2_ at 400 *μ*M caused a reduction of 55.1% in MMP which was rescued by the different honeybush extracts. The red bar represents the H_2_O_2_-treated cells, and the grey bars represent cells that were pretreated for 24 h with the indicated honeybush extract and then treated for 3 h with H_2_O_2_. Values represent as the mean ± SEM of three independent experiments and were normalized on the untreated group (=100%). One-way ANOVA and post hoc Dunnett's multiple comparison test versus (a) untreated (CTRL) or (b) H_2_O_2_. ^∗^*P* < 0.05; ^∗∗^*P* < 0.01; ^∗∗∗^*P* < 0.001.

The aqueous extracts of *C. genistoides* (1 ng/ml) and *C. longifolia* (0.1 and 1 ng/ml) significantly increased MMP up to 24% under physiological conditions after a treatment of 24 h (Figure 5(a)).

H_2_O_2_ at 400 *μ*M caused a significant reduction of 55.1% in MMP which was increased by up to 67.9% by the extracts. In this case, all extracts completely rescued the MMP (Figure 5(b)).

Overall, all extracts acted on the mitochondrial membrane potential by increasing it both under physiological condition and under H_2_O_2_-induced oxidative stress.

### 3.4. Honeybush Extracts Decrease Different Types of ROS under H_2_O_2_-Induced Stress

**Figure 6:**
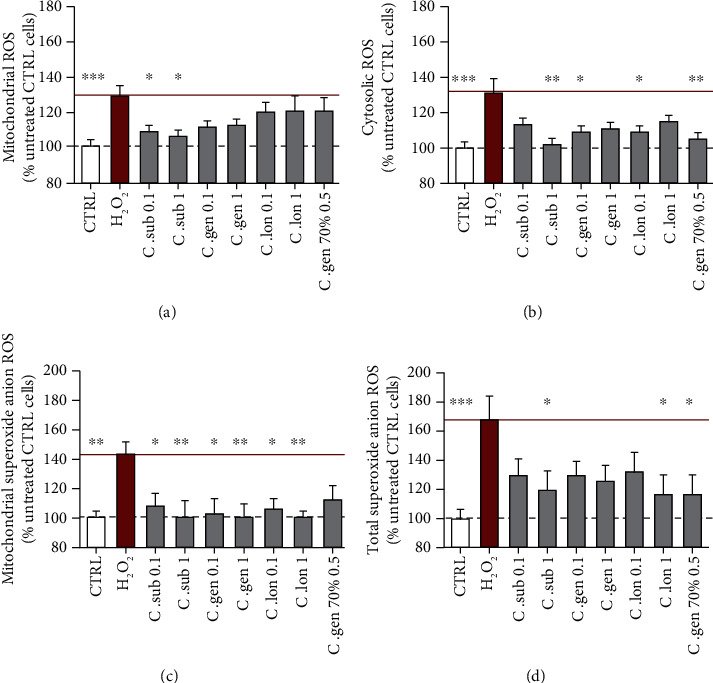
Effect of honeybush extracts on ROS levels under H_2_O_2_-induced oxidative stress. (a) H_2_O_2_ treatment at 400 *μ*M caused an increase of 29.5% in mitochondrial ROS which were detected using the dye DHR. This increase was significantly ameliorated up to 23.1% by *C. subternata* aqueous extract. (b) H_2_O_2_ caused an elevation of 31.2% in cytosolic ROS (detected with the dye DCF). All the extracts could bring the cytosolic ROS levels down, but the ones that reduced them significantly and most effectively were the aqueous extract of *C. subternata* at 1 ng/ml and the 70% ethanolic extract of *C. genistoides* at 0.5 ng/ml. (c) H_2_O_2_ increased the mitochondrial superoxide anion levels by 43%. All the extracts, except the ethanolic extract of *C. genistoides*, significantly reduced the mitochondrial superoxide anion levels. The aqueous extracts of *C. subternata*, *C. genistoides*, and *C. longifolia* at the concentration of 1 ng/ml each completely neutralized the mitochondrial superoxide anion levels. (d) The total superoxide anion levels were elevated by 67.9% in the H_2_O_2_-treated cells. All 4 extracts could ameliorate this increase but only the aqueous extracts of *C. subternata* at 1 ng/ml, *C. longifolia* at 1 ng/ml, and the ethanolic extract of *C. genistoides* at 0.5 ng/ml significantly reduced the superoxide anion levels. The red bar represents the H_2_O_2_-treated cells, and the grey bars represent cells that were pretreated for 24 h with the indicated honeybush extract and then treated for 3 h with H_2_O_2_.Values represent as the mean ± SEM of three independent experiments and were normalized on the untreated group (=100%). One-way ANOVA and post hoc Dunnett's multiple comparison test versus (a) untreated (CTRL) or (b) H_2_O_2_. ^∗^*P* < 0.05; ^∗∗^*P* < 0.01; ^∗∗∗^*P* < 0.001.

H_2_O_2_ at 400 *μ*M caused an increase of 29.5% in mitochondrial ROS which was detected using the dye DHR (dihydrorhodamine 123). This increase was significantly ameliorated up to 23.1% by *C. subternata* water extract. *C. genistoides* also brought the ROS levels down but not significantly (Figure 6(a)).

Cytosolic ROS were detected using the dye DCF (2′,7′-dichlorodihydrofluorescein diacetate). H_2_O_2_ at 400 *μ*M caused an elevation of 31.2%. All extracts lowered cytosolic ROS levels, but the water extract of *C. subternata* at 1 ng/ml (28.9% reduction of cytosolic ROS) and the 70% ethanolic extract of *C. genistoides* at 0.5 ng/ml (26.2% reduction of cytosolic ROS) were the most effective (Figure 6(b)).

H_2_O_2_ at 400 *μ*M increased the mitochondrial superoxide anion levels by 43%. All extracts, except the ethanolic extract of *C. genistoides*, significantly lowered the mitochondrial superoxide anion levels. However, the water extracts of *C. subternata*, *C. genistoides*, and *C. longifolia* at a concentration of 1 ng/ml completely neutralized the mitochondrial superoxide anion levels (reduction of 42%, 42.6%, and 42.6%, respectively) (Figure 6(c)).

The total superoxide anion levels were elevated by 67.9% in the H_2_O_2_-treated cells. All four extracts ameliorated this increase, but only the water extracts of *C. subternata* and *C. longifolia* at 1 ng/ml and the ethanolic extract of *C. genistoides* at 0.5 ng/ml significantly reduced the superoxide anion levels by 48.8%, 50.9%, and 50.3%, respectively (Figure 6(d)).

## 4. Discussion

In this study, we hypothesized that honeybush extracts might exert a beneficial effect on mitochondria of neuronal cells under physiological conditions as well as under oxidative stress due to their phenolic compound content. Neurons have high energy demands and are thus particularly dependent on functional mitochondria. For this reason, we assessed the effects of four different honeybush extracts in a well-characterized neuronal model, the neuroblastoma SH-SY5Y cells. The four extracts were the hot water extracts of *C. subternata*, *C. genistoides*, and *C. longifolia* as well as the 70% ethanolic extract of *C. genistoides*. These extracts were selected after screening the water, 40% ethanolic and 70% ethanolic extracts of these *Cyclopia* species. Hydrogen peroxide (H_2_O_2_) was used as an oxidative stressor as it is one of the most abundant and reactive endogenous ROS.

The beneficial effect of honeybush extracts on mitochondrial functions under physiological conditions and a protective effect under oxidative stress could be demonstrated. The four extracts showed different beneficial properties in different mitochondrial and cellular sites. ATP is the energy that is required for the survival and functionality of cells and especially of neurons which have high energy demands. At the lowest concentrations (0.1-1 ng/ml), all extracts improved the production of ATP under physiological conditions. This increase amounted up to 4% (Figure 1). Also, all extracts were able to significantly increase the ATP levels under H_2_O_2_-induced oxidative stress. This improvement was not a complete rescue but a partial increase of up to 13.5% (Figure 2).

Mitochondrial respiration is an intrinsic function of mitochondria and is essential for the survival of the cells as it results in the production of the majority of ATP. Respiration is taking place at the ETC which is located on the inner mitochondrial membrane (IMM). Glycolysis is the secondary pathway leading to production of ATP. The aqueous extracts of *C. subternata* and *C. genistoides* (both at 1 ng/ml) significantly increased the basal respiration of the mitochondria by up to 40.7%, while those of *C. genistoides* and *C. longifolia* significantly increased glycolysis up to 51.7% under physiological conditions (Figure 3). However, only *C. subternata* aqueous extract (1 ng/ml) could significantly rescue the impaired respiration and only *C. genistoides* aqueous extract (1 ng/ml) could rescue the impaired glycolysis caused by H_2_O_2_ (Figure 4). The aqueous extracts of *C. subternata* and *C. genistoides* specifically acted on respiration. In addition, the *C. subternata* aqueous extract enhanced respiration under oxidative stress. This could be explained by the fact that this extract was the only one that neutralized all four types of tested ROS and particularly the mitochondrial ROS and the mitochondrial superoxide anion which directly affect OXPHOS and respiration (Figure 6). This could be the reason why it was also the only extract to act on respiration under stress.

The aqueous extracts of *C. genistoides* and *C. longifolia* increased the MMP under physiological conditions, while all four extracts completely rescued the MMP under oxidative stress (Figure 5), in addition to partly ameliorating ATP production (Figure 2). During OXPHOS at the ETC of mitochondria, electrons provided by NADH and FADH_2_ are transferred through complexes I-IV. This motion of electrons drives the complexes I, III, and IV to pump protons into the intermembrane space where they are finally used by ATP synthase (complex V) to produce ATP via the phosphorylation of ADP. MMP is an indicator for polarized mitochondrial membranes and therefore an indicator that the pumping of protons in the intermembrane space is not hindered so that they can drive the ATP production by complex V [35, 36]. Amelioration of ATP production under oxidative stress by the extracts could be as a result of their capacity to completely rescue the MMP under oxidative stress and supports this interdependence of MMP and ATP production.

In terms of ROS (Figure 6), pretreatment with the aqueous extract of *C. subternata* (mostly at 1 ng/ml) decreased the four types of tested ROS and it was the only extract of those tested to significantly reduce the mitochondrial ROS (detected with the dye DHR). The result that *C. subternata* extract acted both on mitochondrial superoxide anion levels (detected with the dye MitoSOX) and on all other mitochondrial ROS, such as H_2_O_2_ (detected with the dye DHR), could mean that it either additionally scavenges them or it enhances the activity of the antioxidant defenses that neutralize them (e.g., glutathione, catalase) [21]. The aqueous extract of *C. longifolia* lowered cytosolic ROS, total superoxide anion levels, and mitochondrial superoxide anion levels. The two *C. genistoides* extracts differed, i.e., its aqueous extract neutralized cytosolic ROS and mitochondrial superoxide anion, while its 70% ethanolic extract decreased cytosolic ROS and total superoxide anion levels but had no significant effect on the specific mitochondrial ROS. All extracts had thus a minimizing effect on ROS levels, though at different degrees and on different ROS types (Figure 6). This might be explained by different bioactive components in the specific extract depending on *Cyclopia* species and extraction solvent. While all the water extracts (*C. subternata*, *C. genistoides*, *C. longifolia*) act on mitochondrial superoxide anion levels, the ethanolic extract of *C. genistoides* only affects the cytosolic ROS and total superoxide anion levels. It is assumed that the latter extract acted specifically on cytosolic superoxide anions.

The most beneficial concentrations of the honeybush extracts in this study were found to be as low as 0.1 and 1 ng/ml. Plant extracts are complex mixtures of a multitude of compounds of diverse chemistries and pharmacological activities at different concentrations. The different constituents in the plant extracts could have antagonistic, synergistic, or allosteric effects [37]. For example, an active substance at the higher concentration could have blunted the activity of another bioactive constituent. Possibly, there is one or several constituents that are effective at a low concentration and a gradual increase in concentrations may gradually reduce the efficacy and might explain the observed effect at very low concentrations.

Considering the phenolic profiles of the different extracts, it is clear that no pattern emerged that could explain differential activity. The total polyphenol content often highly correlates with the antioxidant activity *in vitro* but was similar in the aqueous extracts of *C. subternata*, *C. genistoides*, and *C. longifolia* (~26, 25, and 24 g gallic acid equivalents per 100 g extract, respectively). In the ethanolic extract of *C. genistoides*, the phenolic content was slightly increased (~28 g gallic acid equivalents per 100 g extract). However, the phenolic content does not differ substantially between the different extracts to provide an explanation to our findings. In fact, mangiferin, shown to have beneficial effects in *in vitro* and *in vivo* models of neurodegeneration, as well as of oxidative stress [38–43], was lowest in the *C. subternata* water extract and highest in the 70% ethanolic extract of *C. genistoides*. According to these studies, we expected that the *C. genistoides* 70% ethanolic extract would exert the most potent neuroprotective properties, while the aqueous extract of *C. subternata* would exert the least. Interestingly, the results of our experiments proved our assumption wrong as the opposite effect was observed with the aqueous extract of *C. subternata* being the most beneficial extract. A closer observation at the composition of the extracts (Table 1) reveals that the aqueous extract of *C. subternata* contains higher concentrations of flavones and dihydrochalcones. Scolymoside, present in the highest concentration in the *C. subternata* water extract and absent in detectable quantities in the two *C. genistoides* extracts, is a glycoside of luteolin, a flavone aglycone demonstrated to inhibit the production of neuronal mitochondrial superoxide anion O_2_^·–^ [44]. While glycosylation of position C-7 of the A-ring of the flavonoid structure as for scolymoside would decrease its radical scavenging potency compared to luteolin, it does not abolish the activity [45]. Dihydrochalcones related to those in *C. subternata* not only act as radical scavengers [46] but also demonstrated neuroprotective effects [47, 48]. The flavanone, hesperidin, present in the highest level in the 70% ethanolic extract of *C. genistoides* could alleviate oxidative stress [49] and act as neuroprotective agent, amongst others by enhancing endogenous antioxidant defense functions [50].

Regarding the bioavailability of the plant extract, it depends on the bioavailability of the single compounds contained in each extract. Extracts from different honeybush species vary in chemical composition. However, the main active constituents of honeybush have been reported to be mangiferin and hesperidin and there are some data available with regard to their bioavailability and their ability to cross the blood-brain barrier (BBB). Of note, trace amounts of mangiferin were found in the rat brain after an acute oral treatment with a single dose of a plant extract containing mangiferin indicating that the compound can cross the BBB [51], whereas in another study, mangiferin was not detected in the brain of rats after a single dose via intraperitoneal administration [52]. However, one has to take into consideration that different assays of different sensitivities were used in the two studies. In the study from Li et al. (2008), a validated highly sensitive HPLC method was developed and applied to detect mangiferin after a single oral dose of *Rhizoma Anemarrhenae* extract, while in the study of Zajac et al. (2012), a much less sensitive detection method (a simple TLC method) was used. Similarly, the bioavailability of the therapeutically active constituents of *Ginkgo biloba* extract (GBE) in the brain was formerly questioned until recent studies demonstrated the distribution of GBE in the brain of rats after single and repeated oral administration of GBE [53, 54]. The compounds in this case were also successfully detected with an HPLC method. Hesperidin or its aglycone hesperetin seems to be able to traverse the BBB and directly exerts their neuroprotective effect in the brain [55–57].

Furthermore, bioavailability in the brain might be affected by the route of administration and by whether the pure compound is administered or contained in a plant extract but we can assume that mangiferin and hesperidin exert neuroprotective effects on the brain and peripheral neurons.

To sum up, the results obtained from this study indicate that *C. subternata* aqueous extract is the most effective in enhancing mitochondrial functions especially under oxidative stress situations. It was the only one to act on respiration under oxidative stress and the only one to lower all four types of ROS measured in this study. These findings are particularly relevant for the establishment of honeybush tea as nutraceutical as the species that is mostly cultivated for the production of the tea is currently *C. subternata*. The other two aqueous extracts (*C. genistoides* and *C. longifolia*) also exert a beneficial effect. *C. genistoides* acted more on respiration under physiological conditions, while *C. longifolia* was more effective in neutralizing ROS (active against three types of ROS). Interestingly, in the tea industry, honeybush tea is often prepared after blending of different species. Therefore, evaluating the activity of a mixture of different species extracts will be very interesting.

## 5. Conclusion

In this study, the effects of honeybush extracts on enhancing mitochondrial and neuronal functions and on preventing the detrimental effects of oxidative stress were examined. The aqueous extract of *C. subternata* was superior to the other extracts in increasing mitochondrial functions and bioenergetics, especially under H_2_O_2_-induced oxidative stress. The aqueous extracts of *C. genistoides* and *C. longifolia* came next in terms of efficacy on mitochondrial functions. Lower extract concentrations (0.1-1 ng/ml) were also more effective. Overall, our data are in line with existing literature reporting an antioxidant effect of honeybush [19–22]. However, the effects of honeybush extracts on neuronal cells and specifically on mitochondrial function have been investigated here for the first time. Further research is ongoing by our team in order to study more in depth the effect of honeybush in combatting stress and in enhancing neuronal function. These findings make honeybush a potential candidate for prevention of oxidative stress, laying the foundation for further research aimed at the development of a condition-specific nutraceutical.

## Figures and Tables

**Table 1:** Phenolic composition (g/100 g extract) of aqueous extracts of *Cyclopia subternata*, *Cyclopia longifolia*, and *Cyclopia genistoides* and a 70% ethanolic extract of *C. genistoides.*

Compounds	*C. subternata*	*C. longifolia*	*C. genistoides*	
	Water	Water	Water	70% EtOH
Benzophenones				
Maclurin-di-O,C-hexoside (MDH)^a^	nd	nd	0.079	0.061
3-*β*-D-Glucopyranosyl-4-O-*β*-D-glucopyranosyliriflophenone (IDG)	1.67	0.700	1.78	1.41
3-*β*-D-Glucopyranosylmaclurin (MMG)	nd	nd	0.400	0.373
3-*β*-D-Glucopyranosyliriflophenone (IMG)	0.536	0.076	1.52	1.12
Total benzophenones	2.21	0.776	3.77	2.97
Xanthones				
Tetrahydroxyxanthone-di-*O*,*C*-hexoside A (THXA)^b^	nq	0.168	nq	nq
Tetrahydroxyxanthone-di-*O*,*C*-hexoside B (THXB)^b^	nq	0.133	nq	nq
Mangiferin	1.16	6.38	6.86	9.66
Isomangiferin	0.458	1.84	1.97	2.36
Total xanthones	1.62	8.53	8.83	12.0
Flavones				
Vicenin-2	0.182	0.192	0.498	0.524
Scolymoside	1.84	0.497	nq	nq
Total flavones	2.02	0.690	0.498	0.524
Dihydrochalcones				
3-Hydroxyphloretin-di-*C*-hexoside (HPDH)^c^	0.458	nq	nq	nq
3′,5′-Di-*β-*D-glucopyranosylphloretin (PDG)	1.22	nq	nq	nq
Total dihydrochalcones	1.67	0.000	0.000	0.000
Flavanones				
Eriodictyol-*O*-hexoside-*O*-deoxyhexoside (EHD)^d^	nd	0.186	0.297	0.195
(2*R*)-5-*O*-[*α*-L-Rhamnopyranosyl-(1 → 2)-*β*-D-glucopyranosyl]naringenin (2RNAR)^e^	nd	0.028	0.146	0.051
(2*S*)-5-*O*-[*α*-L-Rhamnopyranosyl-(1 → 2)-*β*-D-glucopyranosyl]naringenin (2SNAR)	nd	0.087	0.397	0.444
Eriocitrin	0.536	0.310	nq	nq
Hesperidin	1.43	0.839	0.988	1.73
Total flavanones	1.96	1.45	1.83	2.42
Total quantified phenolics	9.48	11.5	14.9	17.9
Total polyphenols (Folin-Ciocalteau)^f^	25.5	23.7	25.3	27.8

^a^Expressed as MMG equivalents. ^b^Expressed as mangiferin equivalents. ^c^Expressed as PDG equivalents. ^d^Expressed as eriocitrin equivalents. ^e^Expressed as 2SNAR equivalents. ^f^Expressed as g gallic acid equivalents/100 g extract. nd: not detected using LC-MS; nq: present in extract, but not quantified due to coelution of very low content.

## Data Availability

The data used to support the findings of this study are available from the corresponding author upon request.

## Abbreviations

ADP: Adenosine diphosphate
ATP: Adenosine triphosphate
BBB: Blood-brain barrier
DCF: 2′,7′-Dichlorodihydrofluorescein diacetate
DHE: Dihydroethidium
DHR: Dihydrorhodamine 123
DMEM: Dulbecco's modified Eagle medium
DMSO: Dimethyl sulfoxide
ECAR: Extracellular acidification rate
ESI: Electrospray ionization
ETC: Electron transport chain
FADH_2_: Reduced form of flavin adenine dinucleotide (FAD)
FCCP: Carbonyl cyanide-4-(trifluoromethoxy)phenylhydrazone
FCS: Fetal calf serum
GBE: *Ginkgo biloba* extract
GSH: Glutathione(*γ*-glutamylcysteinylglycine)
GSH: GSSG: Reduced glutathione to oxidised glutathione ratio
HBSS: Hanks' balanced salt solution
HPLC-DAD: High-performance liquid chromatography with diode array detector
IMM: Inner mitochondrial membrane
LC-MS: Liquid chromatography-mass spectrometry
MMP: Mitochondrial membrane potential
MS/MS: Tandem mass spectrometry
MtDNA: Mitochondrial DNA
NADH: Reduced form of nicotinamide adenine dinucleotide
OCR: Oxygen consumption rate
OXPHOS: Oxidative phosphorylation
PBS: Phosphate-buffered saline
Q-TOF: Synapt G2 quadrupole time-of-flight
ROS: Reactive oxygen species
SEM: Standard error of the mean
SH-SY5Y: Human neuroblastoma cell line
TMRM: Tetramethylrhodamine methyl ester
UPLC: Ultra-performance liquid chromatography
UV-Vis: Ultraviolet-visible.
